# Supplementary material for: Epidemiology and Outcome of Primary Central Nervous System Tumors Treated at King Hussein Cancer Center
Source: Cancers (Basel). 2025 Feb 9;17(4):590. doi: 10.3390/cancers17040590 (PMC11852727; doi:10.3390/cancers17040590)
Supplement: Supplementary file 1 [file cancers-17-00590-s001.zip › cancers-3368131-supplementary.pdf]

Supplementary Tables

Supplementary Table S1

WHO Group	WHO entity	WHO sub-entity	N (%)
Choroid plexus tumors	Atypical choroid plexus papilloma	Atypical choroid plexus papilloma	8 (44.44%)
Choroid plexus tumors	Choroid plexus carcinoma	Choroid plexus carcinoma	6 (33.33%)
Choroid plexus tumors	Choroid plexus papilloma	Choroid plexus papilloma	4 (22.22%)
Embryonal tumors	Atypical teratoid/rhabdoid tumor	Atypical teratoid/rhabdoid tumor	12 (5.13%)
Embryonal tumors	CNS embryonal tumor	CNS embryonal tumor	15 (6.41%)
Embryonal tumors	CNS embryonal tumor	CNS neuroblastoma	2 (0.85%)
Embryonal tumors	Embryonal tumor with multilayered rosettes	Embryonal tumor with multilayered rosettes	5 (2.14%)
Embryonal tumors	Embryonal tumor with multilayered rosettes	Medulloepithelioma	1 (0.43%)
Embryonal tumors	Medulloblastoma	Medulloblastoma	199 (85.04%)
Germ cell tumors	Choriocarcinoma	Choriocarcinoma	1 (5.88%)
Germ cell tumors	Germinoma	Germinoma	15 (88.24%)
Germ cell tumors	Teratoma	Teratoma	1 (5.88%)
Gliomas, glioneuronal tumors, and neuronal tumors	Adult-type diffuse gliomas	Astrocytoma, grade-3	94 (7.83%)
Gliomas, glioneuronal tumors, and neuronal tumors	Adult-type diffuse gliomas	*Anaplastic oligoastrocytoma	9 (0.75%)
Gliomas, glioneuronal tumors, and neuronal tumors	Adult-type diffuse gliomas	Oligodendroglioma, grade-3	33 (2.75%)
Gliomas, glioneuronal tumors, and neuronal tumors	Adult-type diffuse gliomas	Astrocytoma, grade 2	88 (7.33%)
Gliomas, glioneuronal tumors, and neuronal tumors	Adult-type diffuse gliomas	Glioblastoma	439 (36.58%)
Gliomas, glioneuronal tumors, and neuronal tumors	Adult-type diffuse gliomas	*Oligoastrocytoma	4 (0.33%)
Gliomas, glioneuronal tumors, and neuronal tumors	Adult-type diffuse gliomas	Oligodendroglioma, grade-2	42 (3.5%)
Gliomas, glioneuronal tumors, and neuronal tumors	Circumscribed astrocytic glioma	Pleomorphic xanthoastrocytoma, grade-3	3 (0.25%)
Gliomas, glioneuronal tumors, and neuronal tumors	Circumscribed astrocytic glioma	Astroblastoma	1 (0.08%)
Gliomas, glioneuronal tumors, and neuronal tumors	Circumscribed astrocytic glioma	Pilocytic astrocytoma	166 (13.83%)
Gliomas, glioneuronal tumors, and neuronal tumors	Circumscribed astrocytic glioma	Pleomorphic xanthoastrocytoma	11 (0.92%)
Gliomas, glioneuronal tumors, and neuronal tumors	Circumscribed astrocytic glioma	Subependymal giant cell astrocytoma	2 (0.17%)
Gliomas, glioneuronal tumors, and neuronal tumors	Ependymal tumors	Ependymoma, grade-3	32 (2.67%)
Gliomas, glioneuronal tumors, and neuronal tumors	Ependymal tumors	Ependymoma, grade-2	50 (4.17%)

Gliomas, glioneuronal tumors, and neuronal tumors	Ependymal tumors	Myxopapillary ependymoma	6 (0.5%)
Gliomas, glioneuronal tumors, and neuronal tumors	Ependymal tumors	Subependymoma	1 (0.08%)
Gliomas, glioneuronal tumors, and neuronal tumors	Glioneuronal and neuronal tumors	Ganglioglioma, grade-3	5 (0.42%)
Gliomas, glioneuronal tumors, and neuronal tumors	Glioneuronal and neuronal tumors	Central neurocytoma	11 (0.92%)
Gliomas, glioneuronal tumors, and neuronal tumors	Glioneuronal and neuronal tumors	Desmoplastic infantile astrocytoma and ganglioglioma	2 (0.17%)
Gliomas, glioneuronal tumors, and neuronal tumors	Glioneuronal and neuronal tumors	Dysembryoplastic neuroepithelial tumor	6 (0.5%)
Gliomas, glioneuronal tumors, and neuronal tumors	Glioneuronal and neuronal tumors	Dysplastic cerebellar gangliocytoma (Lhermitte–Duclos disease)	1 (0.08%)
Gliomas, glioneuronal tumors, and neuronal tumors	Glioneuronal and neuronal tumors	Ganglioglioma	16 (1.33%)
Gliomas, glioneuronal tumors, and neuronal tumors	Glioneuronal and neuronal tumors	Papillary glioneuronal tumor	2 (0.17%)
Gliomas, glioneuronal tumors, and neuronal tumors	Pediatric-type diffuse high-grade gliomas	Astrocytoma, grade-3	16 (1.33%)
Gliomas, glioneuronal tumors, and neuronal tumors	Pediatric-type diffuse high-grade gliomas	*Oligoastrocytoma	1 (0.08%)
Gliomas, glioneuronal tumors, and neuronal tumors	Pediatric-type diffuse high-grade gliomas	Oligodendroglioma, grade-3	1 (0.08%)
Gliomas, glioneuronal tumors, and neuronal tumors	Pediatric-type diffuse high-grade gliomas	Diffuse midline glioma	100 (8.33%)
Gliomas, glioneuronal tumors, and neuronal tumors	Pediatric-type diffuse high-grade gliomas	Glioblastoma	44 (3.67%)
Gliomas, glioneuronal tumors, and neuronal tumors	Pediatric-type diffuse low-grade gliomas	Astrocytoma, grade 2	13 (1.08%)
Gliomas, glioneuronal tumors, and neuronal tumors	Pediatric-type diffuse low-grade gliomas	Oligodendroglioma, grade-2	1 (0.08%)
Hematolymphoid tumors	Diffuse large B-cell lymphoma of the CNS	Diffuse large B-cell lymphoma of the CNS	38 (100%)
Melanocytic tumors	Meningeal melanoma	Meningeal melanoma	2 (100%)
Meningiomas	Anaplastic meningioma	Anaplastic meningioma	17 (6.51%)
Meningiomas	Anaplastic meningioma	Papillary meningioma	1 (0.38%)
Meningiomas	Atypical meningioma	Atypical meningioma	25 (9.58%)
Meningiomas	Atypical meningioma	Clear cell meningioma	5 (1.92%)
Meningiomas	Meningioma	Meningioma	148 (56.7%)
Meningiomas	Meningioma	Meningothelial meningioma	48 (18.39%)
Meningiomas	Meningioma	Psammomatous meningioma	6 (2.3%)
Meningiomas	Meningioma	Transitional meningioma	11 (4.21%)
Mesenchymal, non-meningothelial tumors	Hemangioblastoma	Hemangioblastoma	16 (38.1%)
Mesenchymal, non-meningothelial tumors	Hemangioma	Hemangioma	13 (30.95%)

Mesenchymal, non-meningothelial tumors	Lipoma	Lipoma	2 (4.76%)
Mesenchymal, non-meningothelial tumors	Solitary fibrous tumor/hemangiopericytoma	Solitary fibrous tumor/hemangiopericytoma	11 (26.19%)
Miscellaneous	Dermoid cyst	Dermoid cyst	2 (1.26%)
Miscellaneous	`	Pituitary Neuroendocrine Tumors (PitNET)	157 (98.74%)
Tumors of the cranial and paraspinal nerves	Malignant peripheral nerve sheath tumor	Malignant peripheral nerve sheath tumor	7 (12.07%)
Tumors of the cranial and paraspinal nerves	Neurofibroma	Neurofibroma	51 (87.93%)
Tumors of the pineal region	Papillary tumor of the pineal region	Papillary tumor of the pineal region	1 (14.29%)
Tumors of the pineal region	Pineoblastoma	Pineoblastoma	6 (85.71%)
Tumors of the sellar region	Craniopharyngioma	Craniopharyngioma	56 (96.55%)
Tumors of the sellar region	Pituicytoma	Pituicytoma	2 (3.45%)
* ; Oligoastrocytoma is an obsolete diagnosis, however, a few cases could not be reclassified as per the WHO 2021 CNS Tumor Classification			

Table S1: Distribution of Primary Central Nervous System (CNS) Tumors by WHO Group, Entity, and Sub-Entity in Patients Managed at King Hussein Cancer Center (KHCC) Between July 2003 and June 2019.

Supplementary Table S2

Characteristic	1-year	2-year	5-year
**WHO Group**			
Choroid plexus tumors	100% (100%, 100%)	89% (75%, 100%)	75% (57%, 100%)
Embryonal tumors	83% (78%, 88%)	72% (66%, 78%)	61% (55%, 68%)
Germ cell tumors	100% (100%, 100%)	94% (84%, 100%)	88% (74%, 100%)
Gliomas, glioneuronal tumors, and neuronal tumors	70% (67%, 72%)	53% (50%, 56%)	41% (39%, 45%)
Hematolymphoid tumors	63% (50%, 81%)	43% (30%, 63%)	22% (11%, 45%)
Melanocytic tumors	50% (13%, 100%)	50% (13%, 100%)	— (—, —)
Meningiomas	97% (95%, 99%)	94% (92%, 97%)	84% (79%, 89%)
Mesenchymal, non-meningothelial tumors	88% (79%, 98%)	85% (75%, 97%)	82% (70%, 95%)
Miscellaneous	98% (96%, 100%)	97% (95%, 100%)	93% (88%, 98%)
Tumors of the cranial and paraspinal nerves	100% (100%, 100%)	98% (95%, 100%)	92% (84%, 100%)
Tumors of the pineal region	86% (63%, 100%)	43% (18%, 100%)	43% (18%, 100%)
Tumors of the sellar region	93% (87%, 100%)	90% (82%, 98%)	80% (70%, 92%)
**WHO Entity**			
Adult-type diffuse gliomas	64% (61%, 68%)	42% (39%, 46%)	28% (24%, 31%)
Anaplastic meningioma	89% (75%, 100%)	89% (75%, 100%)	62% (42%, 92%)
Atypical choroid plexus papilloma	100% (100%, 100%)	100% (100%, 100%)	100% (100%, 100%)
Atypical meningioma	97% (90%, 100%)	93% (85%, 100%)	85% (73%, 100%)
Atypical teratoid/rhabdoid tumor	25% (9.4%, 67%)	17% (4.7%, 59%)	— (—, —)
Choriocarcinoma	100% (100%, 100%)	100% (100%, 100%)	— (—, —)
Choroid plexus carcinoma	100% (100%, 100%)	67% (38%, 100%)	22% (4.1%, 100%)
Choroid plexus papilloma	100% (100%, 100%)	100% (100%, 100%)	100% (100%, 100%)
Circumscribed astrocytic glioma	95% (91%, 98%)	92% (88%, 96%)	91% (86%, 95%)
CNS embryonal tumor	88% (74%, 100%)	47% (28%, 78%)	24% (10.0%, 55%)
Craniopharyngioma	93% (86%, 100%)	89% (81%, 98%)	80% (69%, 92%)
Dermoid cyst	100% (100%, 100%)	100% (100%, 100%)	100% (100%, 100%)
Diffuse large B-cell lymphoma of the CNS	63% (50%, 81%)	43% (30%, 63%)	22% (11%, 45%)

Embryonal tumor with multilayered rosettes	33% (11%, 100%)	17% (2.8%, 100%)	— (—, —)
Ependymal tumors	93% (88%, 99%)	85% (78%, 93%)	69% (59%, 80%)
Germinoma	100% (100%, 100%)	93% (82%, 100%)	93% (82%, 100%)
Glioneuronal and neuronal tumors	91% (82%, 100%)	91% (82%, 100%)	87% (78%, 99%)
Hemangioblastoma	94% (83%, 100%)	87% (71%, 100%)	78% (58%, 100%)
Hemangioma	92% (79%, 100%)	92% (79%, 100%)	92% (79%, 100%)
Lipoma	50% (13%, 100%)	50% (13%, 100%)	50% (13%, 100%)
Malignant peripheral nerve sheath tumor	100% (100%, 100%)	100% (100%, 100%)	100% (100%, 100%)
Medulloblastoma	87% (83%, 92%)	79% (73%, 85%)	69% (63%, 76%)
Meningeal melanoma	50% (13%, 100%)	50% (13%, 100%)	— (—, —)
Meningioma	98% (96%, 100%)	95% (92%, 98%)	86% (80%, 91%)
Neurofibroma	100% (100%, 100%)	98% (94%, 100%)	90% (82%, 100%)
Papillary tumor of the pineal region	100% (100%, 100%)	— (—, —)	— (—, —)
Pediatric-type diffuse high-grade gliomas	46% (39%, 55%)	26% (20%, 34%)	18% (13%, 26%)
Pediatric-type diffuse low-grade gliomas	79% (60%, 100%)	71% (51%, 99%)	63% (42%, 95%)
Pineoblastoma	83% (58%, 100%)	50% (22%, 100%)	50% (22%, 100%)
Pituicytoma	100% (100%, 100%)	100% (100%, 100%)	100% (100%, 100%)
Pituitary Neuroendocrine Tumors (PitNET)	98% (96%, 100%)	97% (95%, 100%)	93% (88%, 98%)
Solitary fibrous tumor/hemangiopericytoma	82% (62%, 100%)	82% (62%, 100%)	82% (62%, 100%)
Teratoma	100% (100%, 100%)	100% (100%, 100%)	— (—, —)

Table S2: 1-Year, 2-Year, and 5-Year Overall Survival Rates by WHO Group and Entity for Patients with Primary Central Nervous System (CNS) Tumors at KHCC Between July 2003 and June 2019.

Supplementary Table S3

	<2012			>2012		
**Characteristic**	**Time 12**	**Time 24**	**Time 60**	**Time 12**	**Time 24**	**Time 60**
**WHO Group**						
Choroid plexus tumors	100% (100%, 100%)	100% (100%, 100%)	100% (100%, 100%)	NA	NA	NA
Embryonal tumors	100% (100%, 100%)	87% (75%, 100%)	67% (52%, 86%)	97% (91%, 100%)	97% (91%, 100%)	73% (57%, 92%)
Germ cell tumors	100% (100%, 100%)	100% (100%, 100%)	100% (100%, 100%)	100% (100%, 100%)	100% (100%, 100%)	100% (100%, 100%)
Gliomas, glioneuronal tumors, and neuronal tumors	66% (62%, 72%)	45% (40%, 50%)	31% (27%, 37%)	67% (63%, 72%)	48% (44%, 53%)	33% (29%, 39%)
Hematolymphoid tumors	69% (48%, 99%)	54% (33%, 89%)	15% (4.3%, 55%)	61% (44%, 84%)	41% (24%, 68%)	41% (24%, 68%)
Melanocytic tumors	NA	NA	NA	0% (—, —)	0% (—, —)	0% (—, —)
Meningiomas	99% (96%, 100%)	95% (91%, 100%)	83% (75%, 92%)	97% (94%, 100%)	94% (91%, 98%)	84% (78%, 91%)
Mesenchymal, non-meningothelial tumors	100% (100%, 100%)	100% (100%, 100%)	100% (100%, 100%)	86% (74%, 100%)	81% (68%, 98%)	74% (57%, 96%)
Miscellaneous	98% (95%, 100%)	97% (92%, 100%)	90% (83%, 98%)	98% (95%, 100%)	98% (95%, 100%)	95% (90%, 100%)
Tumors of the cranial and paraspinal nerves	100% (100%, 100%)	100% (100%, 100%)	86% (72%, 100%)	100% (100%, 100%)	96% (90%, 100%)	96% (90%, 100%)
Tumors of the pineal region	67% (30%, 100%)	33% (6.7%, 100%)	33% (6.7%, 100%)	NA	NA	NA
Tumors of the sellar region	100% (100%, 100%)	100% (100%, 100%)	100% (100%, 100%)	93% (80%, 100%)	93% (80%, 100%)	73% (51%, 100%)
**Gliomas, glioneuronal tumors, and neuronal tumors**						
Adult-type diffuse gliomas	64% (59%, 70%)	41% (36%, 47%)	27% (22%, 32%)	64% (60%, 69%)	43% (39%, 49%)	28% (23%, 34%)
Circumscribed astrocytic glioma	80% (52%, 100%)	80% (52%, 100%)	80% (52%, 100%)	81% (66%, 100%)	81% (66%, 100%)	81% (66%, 100%)

Ependymal tumors	100% (100%, 100%)	94% (84%, 100%)	83% (68%, 100%)	94% (83%, 100%)	79% (60%, 100%)	56% (33%, 97%)
Glioneuronal and neuronal tumors	88% (67%, 100%)	88% (67%, 100%)	88% (67%, 100%)	88% (74%, 100%)	88% (74%, 100%)	79% (61%, 100%)
Pediatric-type diffuse high-grade gliomas	57% (30%, 100%)	14% (2.3%, 88%)	14% (2.3%, 88%)	78% (61%, 100%)	59% (39%, 88%)	47% (26%, 85%)
**Meningiomas**						
Anaplastic meningioma	91% (75%, 100%)	91% (75%, 100%)	64% (41%, 99%)	100% (100%, 100%)	100% (100%, 100%)	50% (13%, 100%)
Atypical meningioma	100% (100%, 100%)	92% (79%, 100%)	92% (79%, 100%)	92% (79%, 100%)	92% (79%, 100%)	69% (45%, 100%)
Meningioma	100% (100%, 100%)	97% (92%, 100%)	85% (76%, 94%)	97% (95%, 100%)	94% (90%, 98%)	86% (80%, 93%)

Supplementary Table S4

	<2012			>2012		
**Characteristic**	**Time 12**	**Time 24**	**Time 60**	**Time 12**	**Time 24**	**Time 60**
**WHO Group**						
Choroid plexus tumors	75% (43%, 100%)	75% (43%, 100%)	50% (19%, 100%)	100% (100%, 100%)	91% (75%, 100%)	80% (58%, 100%)
Embryonal tumors	77% (68%, 87%)	61% (51%, 73%)	53% (43%, 66%)	78% (70%, 87%)	68% (59%, 78%)	64% (54%, 75%)
Germ cell tumors	100% (100%, 100%)	100% (100%, 100%)	100% (100%, 100%)	100% (100%, 100%)	86% (63%, 100%)	71% (45%, 100%)
Gliomas, glioneuronal tumors, and neuronal tumors	78% (72%, 86%)	66% (59%, 75%)	57% (49%, 66%)	74% (68%, 79%)	66% (60%, 72%)	62% (56%, 69%)
Hematolymphoid tumors	100% (100%, 100%)	0% (—, —)	0% (—, —)	0% (—, —)	0% (—, —)	0% (—, —)
Melanocytic tumors	NA	NA	NA	100% (100%, 100%)	100% (100%, 100%)	100% (100%, 100%)
Meningiomas	50% (13%, 100%)	50% (13%, 100%)	50% (13%, 100%)	100% (100%, 100%)	100% (100%, 100%)	100% (100%, 100%)
Mesenchymal, non-meningothelial tumors	0% (—, —)	0% (—, —)	0% (—, —)	100% (100%, 100%)	100% (100%, 100%)	100% (100%, 100%)
Miscellaneous	100% (100%, 100%)	100% (100%, 100%)	100% (100%, 100%)	100% (100%, 100%)	100% (100%, 100%)	100% (100%, 100%)
Tumors of the cranial and paraspinal nerves	100% (100%, 100%)	100% (100%, 100%)	100% (100%, 100%)	100% (100%, 100%)	100% (100%, 100%)	100% (100%, 100%)
Tumors of the pineal region	100% (100%, 100%)	50% (13%, 100%)	50% (13%, 100%)	100% (100%, 100%)	50% (13%, 100%)	50% (13%, 100%)
Tumors of the sellar region	81% (64%, 100%)	81% (64%, 100%)	75% (57%, 100%)	100% (100%, 100%)	87% (72%, 100%)	70% (43%, 100%)

**Gliomas, glioneuronal tumors, and neuronal tumors**						
Circumscribed astrocytic glioma	93% (87%, 100%)	86% (78%, 96%)	85% (76%, 94%)	99% (97%, 100%)	99% (97%, 100%)	97% (93%, 100%)
Ependymal tumors	96% (88%, 100%)	83% (68%, 100%)	52% (35%, 77%)	88% (77%, 100%)	84% (73%, 98%)	80% (67%, 96%)
Glioneuronal and neuronal tumors	100% (100%, 100%)	100% (100%, 100%)	100% (100%, 100%)	93% (80%, 100%)	93% (80%, 100%)	93% (80%, 100%)
Pediatric-type diffuse high-grade gliomas	47% (34%, 64%)	28% (17%, 45%)	16% (8.3%, 32%)	39% (31%, 51%)	21% (14%, 31%)	15% (9.1%, 25%)
Pediatric-type diffuse low-grade gliomas	80% (52%, 100%)	60% (29%, 100%)	60% (29%, 100%)	78% (55%, 100%)	78% (55%, 100%)	65% (39%, 100%)
**Embryonal tumors**						
Atypical teratoid/rhabdoid tumor	14% (2.3%, 88%)	0% (—, —)	0% (—, —)	40% (14%, 100%)	40% (14%, 100%)	40% (14%, 100%)
CNS embryonal tumor	86% (63%, 100%)	29% (8.9%, 92%)	29% (8.9%, 92%)	67% (30%, 100%)	0% (—, —)	0% (—, —)
Embryonal tumor with multilayered rosettes	NA	NA	NA	33% (11%, 100%)	17% (2.8%, 100%)	17% (2.8%, 100%)
Medulloblastoma	83% (74%, 92%)	71% (61%, 84%)	62% (51%, 75%)	84% (77%, 92%)	76% (67%, 86%)	71% (62%, 82%)

Supplementary Figures
Figures

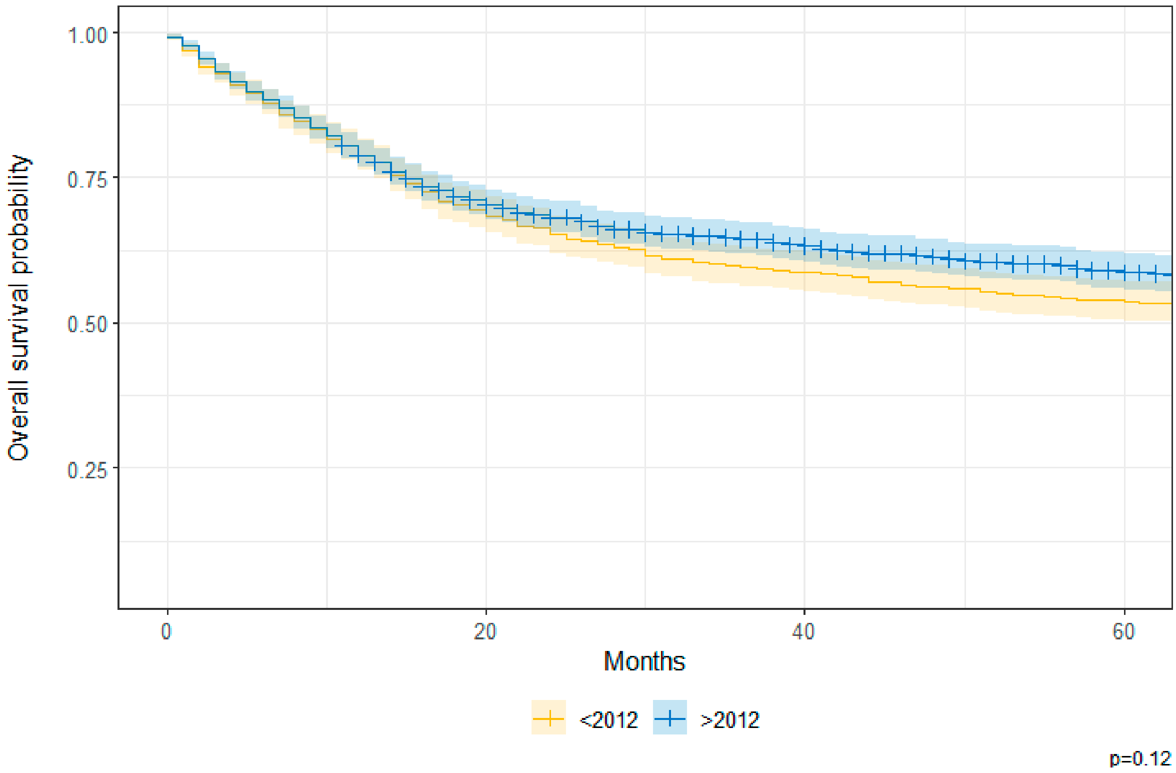

<2012				
At Risk	831	578	489	447
Events	7	262	344	385
>2012				
At Risk	1263	794	514	326
Events	9	369	437	468

Figure S1: Overall Survival Probability for Adult-Type Diffuse Gliomas Diagnosed Before and After 2012.

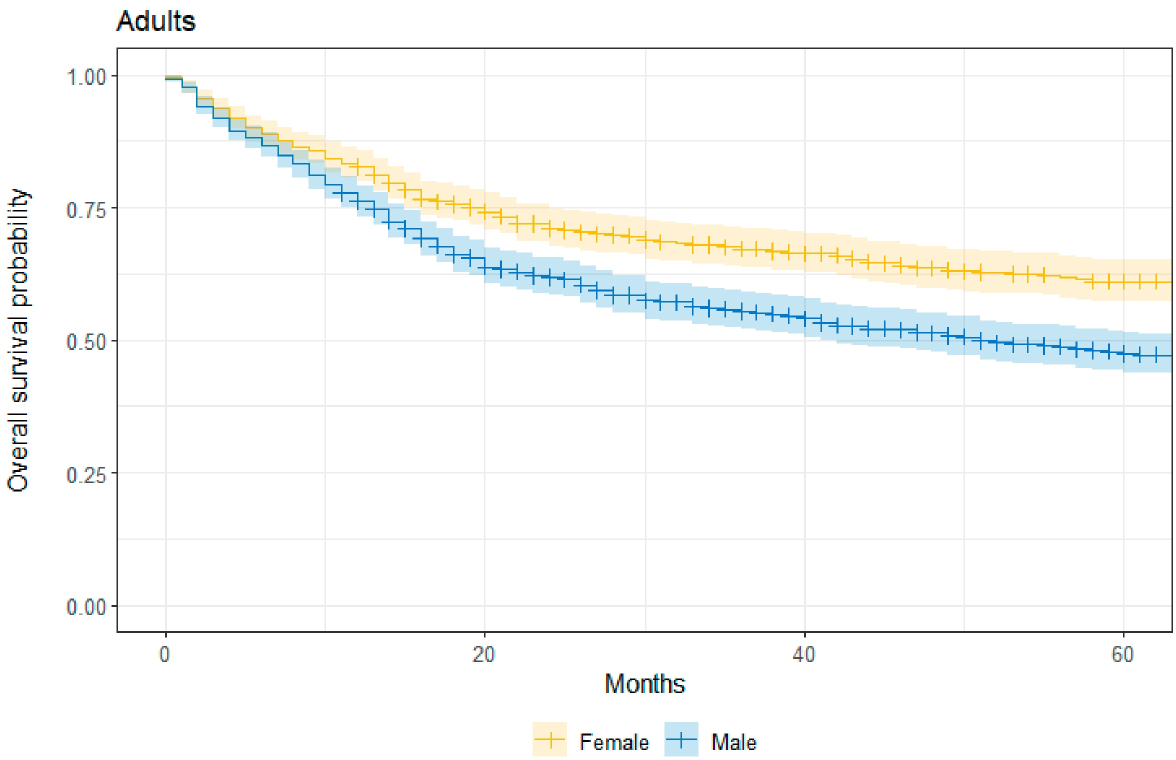

p<0.001

**Female**				
At Risk	653	449	329	253
Events	3	166	209	233
**Male**				
At Risk	789	477	336	250
Events	6	281	346	384

Figure S2: Overall Survival Probability by Gender for Adult Patients with Primary Central Nervous System (CNS) Tumors at KHCC Between July 2003 and June 2019.

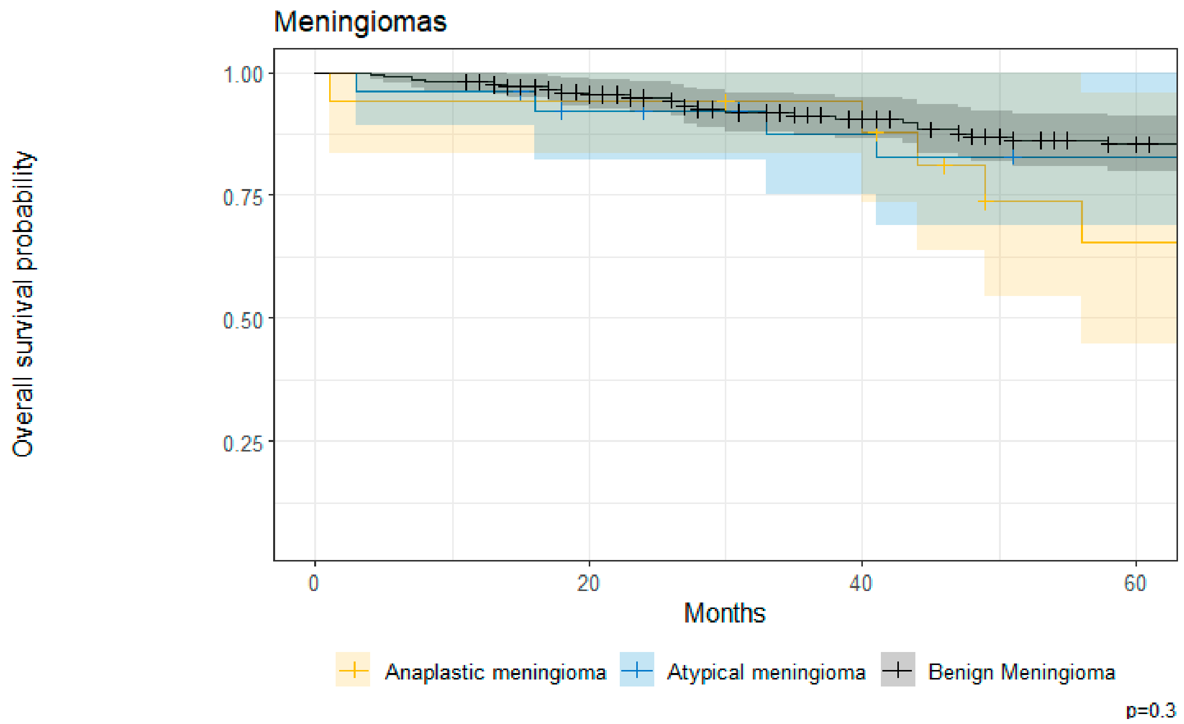

At Risk

	0	20	40	60
Anaplastic meningioma	17	16	15	8
Atypical meningioma	26	22	19	17
Benign Meningioma	207	175	131	104

Events

	0	20	40	60
Anaplastic meningioma	0	1	2	5
Atypical meningioma	0	2	3	4
Benign Meningioma	0	9	17	24

Figure S3: Overall Survival Probability for Patients with Meningiomas (Anaplastic, Atypical, and Meningioma) at KHCC Between July 2003 and June 2019.

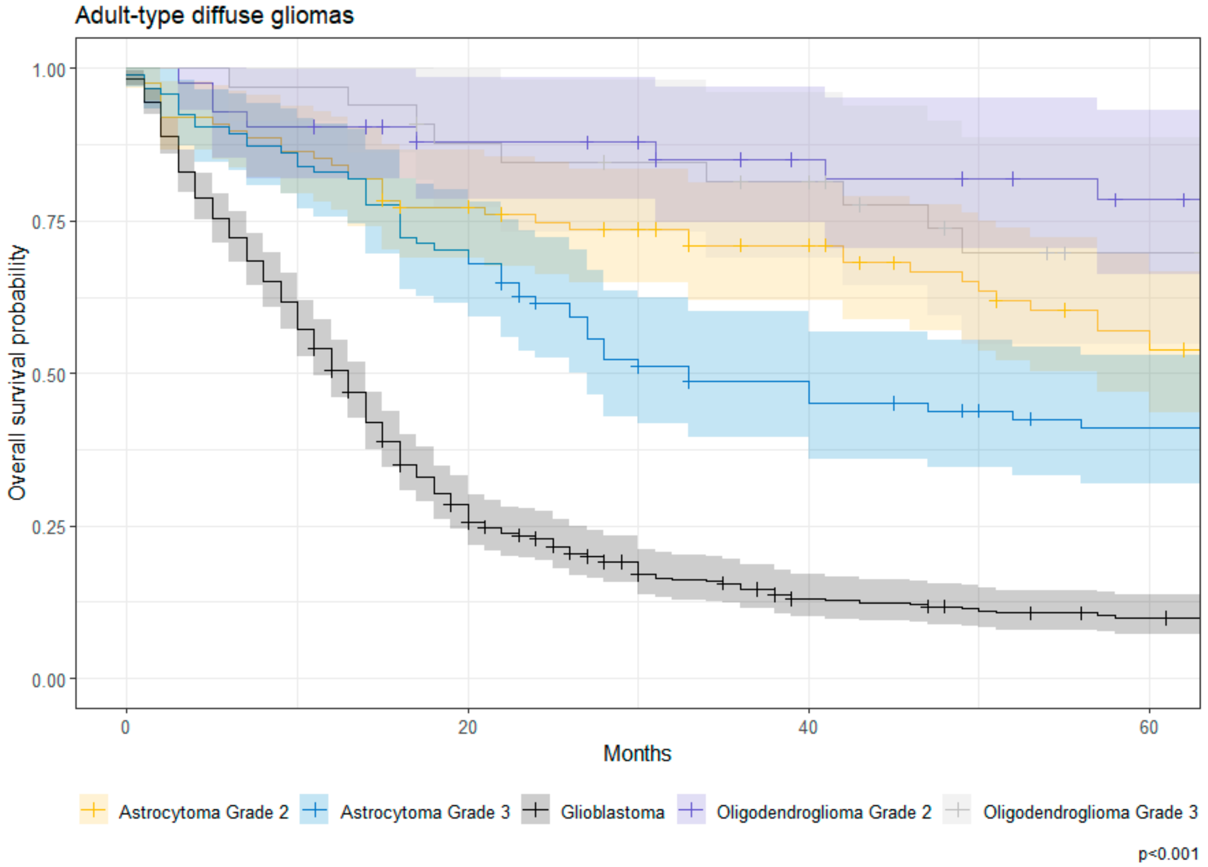

Figure S4: Overall Survival Probability for Patients with Adult-Type Diffuse Gliomas by Subtype at KHCC Between July 2003 and June 2019.

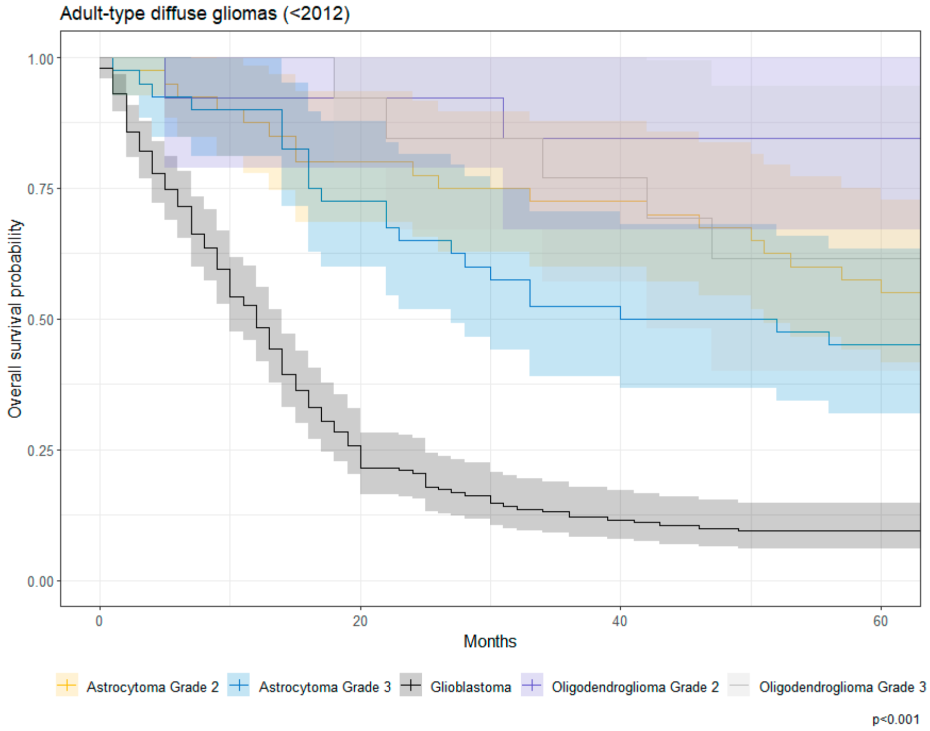

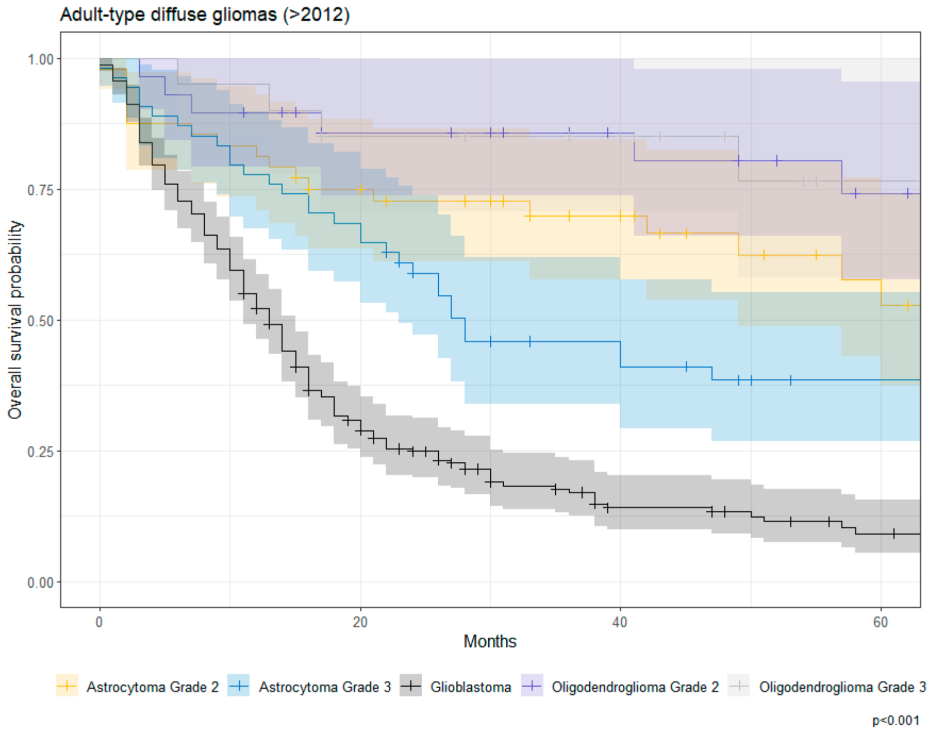

Figure S5: Overall Survival Probability for Adult-Type Diffuse Gliomas by Subtype Diagnosed Before and After 2012.

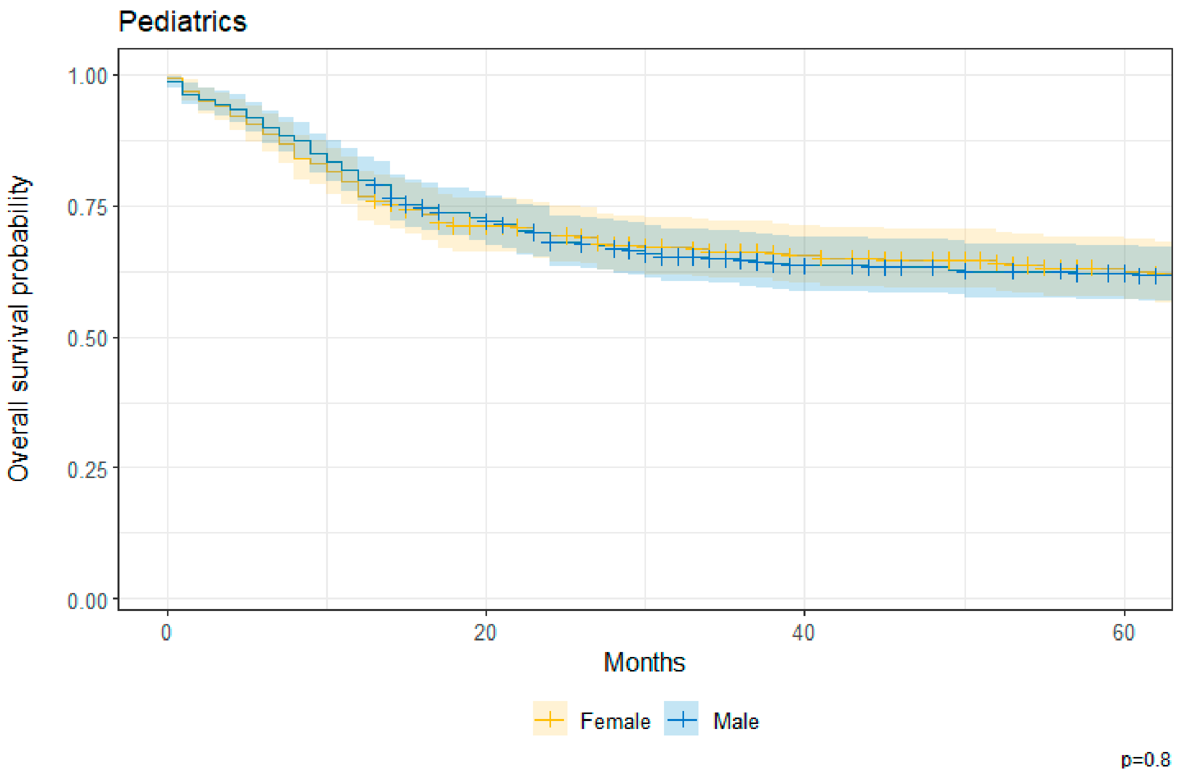

**Female**				
At Risk	296	200	155	111
Events	2	85	100	106
**Male**				
At Risk	356	246	183	159
Events	5	99	126	130

Figure S6: Overall Survival Probability by Gender for Pediatric Patients with Primary Central Nervous System (CNS) Tumors at KHCC Between July 2003 and June 2019.
